# Myoelectric prosthesis hand grasp control following targeted muscle reinnervation in individuals with transradial amputation

**DOI:** 10.1371/journal.pone.0280210

**Published:** 2023-01-26

**Authors:** Ann M. Simon, Kristi L. Turner, Laura A. Miller, Gregory A. Dumanian, Benjamin K. Potter, Mark D. Beachler, Levi J. Hargrove, Todd A. Kuiken

**Affiliations:** 1 Center for Bionic Medicine, Shirley Ryan AbilityLab, Chicago, IL, United States of America; 2 Department of Physical Medicine and Rehabilitation, Northwestern University, Chicago, IL, United States of America; 3 Division of Plastic Surgery, Northwestern Feinberg School of Medicine, Chicago, IL, United States of America; 4 Uniformed Services University–Walter Reed National Military Medical Center Department of Surgery, Bethesda, MD, United States of America; 5 Orthotic & Prosthetic Service, Department of Rehabilitation, Walter Reed National Military Medical Center, Bethesda, MD, United States of America; 6 Department of Biomedical Engineering, Northwestern University, Evanston, IL, United States of America; Szegedi Tudomanyegyetem, HUNGARY

## Abstract

**Background:**

Despite the growing availability of multifunctional prosthetic hands, users’ control and overall functional abilities with these hands remain limited. The combination of pattern recognition control and targeted muscle reinnervation (TMR) surgery, an innovative technique where amputated nerves are transferred to reinnervate new muscle targets in the residual limb, has been used to improve prosthesis control of individuals with more proximal upper limb amputations (i.e., shoulder disarticulation and transhumeral amputation).

**Objective:**

The goal of this study was to determine if prosthesis hand grasp control improves following transradial TMR surgery.

**Methods:**

Eight participants were trained to use a multi-articulating hand prosthesis under myoelectric pattern recognition control. All participated in home usage trials pre- and post-TMR surgery. Upper limb outcome measures were collected following each home trial.

**Results:**

Three outcome measures (Southampton Hand Assessment Procedure, Jebsen-Taylor Hand Function Test, and Box and Blocks Test) improved 9–12 months post-TMR surgery compared with pre-surgery measures. The Assessment of Capacity for Myoelectric Control and Activities Measure for Upper Limb Amputees outcome measures had no difference pre- and post-surgery. An offline electromyography analysis showed a decrease in grip classification error post-TMR surgery compared to pre-TMR surgery. Additionally, a majority of subjects noted qualitative improvements in their residual limb and phantom limb sensations post-TMR.

**Conclusions:**

The potential for TMR surgery to result in more repeatable muscle contractions, possibly due to the reduction in pain levels and/or changes to phantom limb sensations, may increase functional use of many of the clinically available dexterous prosthetic hands.

## Introduction

Transradial amputations account for over 40% of major upper limb amputations [1] and greatly affect an individual’s ability to perform functional tasks in daily life. Due to the amount of dexterity lost in the missing hand, single degree of freedom prosthetic hand components, whether more cosmetically shaped hand and fingers or hook-like terminal devices, are used more as a tool than as a replacement [2]. Two main factors that have limited improved function with transradial prostheses include the lack of robust, natural control of more than one degree of freedom and effective hand prostheses that can provide more of the prehensile functions of the intact hand [3].

The newest generation of prosthetic hand components addresses one of these factors. More anthropometric, multi-articulating hands with variable design tradeoffs [2, 4, 5], present users with a larger set of grasp patterns to hold and manipulate objects of various sizes, shapes, and weights. Control options to switch between grips include smartphone applications, buttons mounted directly to the prosthesis, proximity sensors, IMU gesture-based control, and/or myoelectric control [5–8]. Even with all these options, the cognitive burden remains high and affects users’ ability to quickly and reliably choose an appropriate grip for activities of daily living. Myoelectric pattern recognition is one way to reduce the cognitive burden of prosthesis control [9], whereby residual limb muscle contractions can be used to train an intent recognition system [10–12]. With pattern recognition of hand control, users can make physiologically appropriate muscle contractions to close their phantom hand in various grasp patterns or individual finger motions to control a prosthesis [13–17].

Targeted Muscle Reinnervation (TMR) surgery is an additional significant advancement in the control of multifunction prostheses [18–20]. With TMR surgery, an amputated nerve is transferred to a remaining muscle site in the residual limb and, over the course of several months, the muscle is reinnervated sufficiently that EMG signals can be measured using surface electrodes. Reinnervated muscles, especially for more proximal upper limb amputation levels (i.e., shoulder disarticulations and transhumeral amputations) can provide important additional neural control information for improved myoelectric prosthesis control [11, 18, 21–23]. Because the transferred nerves contain both motor and sensory information, target sensory reinnervation is also possible in the residual limb. To improve sensory outcomes, skin over the targeted muscle is denervated and then sensory nerve fibers in the transferred nerves reinnervate this targeted area of skin. When reinnervated skin is touched the individual feels as if the missing hand is touched [24]; reinnervated skin can sense touch, temperature, pain and vibration [18]. A potential further benefit of TMR surgery is its potential to treat painful neuromas [25]; a recent clinical trial showed TMR surgery improved phantom limb pain and trended toward improved residual limb pain compared to conventional neurectomy [26].

For individuals with a transradial amputation, TMR surgery has the potential to restore data from intrinsic hand muscles. The inclusion of neural information from intrinsic hand muscles has been shown to improve hand grasp and finger movement classification [27]. For individuals with a transradial amputation to benefit from this additional information via the TMR procedure it would require denervation of extrinsic hand muscle motor points, potentially removing intrinsic muscle data. It is currently unknown to what extent individuals with more distal amputation levels can benefit from improved prosthesis control following TMR surgery in the residual forearm.

All intrinsic hand muscles are innervated by the median and ulnar nerves, thus only two nerve transfers are needed to provide more complete neural information for pattern recognition control. Potential target muscle sites in the residual forearm include flexor digitorum superficialis (FDS), flexor digitorum profundus (FDP), or brachioradialis for the median nerve and flexor carpi ulnaris (FCU) or flexor pollicis longus (FPL) for the ulnar nerve [28–30]. These target muscles are superficial and have close proximity to the donor motor nerve. Furthermore, there are other muscles in the forearm that perform similar functions to these muscles so no important function or electromyography (EMG) information should be lost as a result of the transfers.

The goal of this study was to evaluate hand grasp control for individuals with a transradial amputation following TMR surgery. Study objectives were to 1) determine if TMR improved functional outcome measures for individuals with transradial amputations, 2) assess improvement in EMG signals during offline analysis, and 3) evaluate user’s subjective representation of their phantom limbs after TMR.

## Methods

Individuals were recruited from across the US from January 2015 through October 2018. All fittings, surgery, and testing were conducted from September 2015 through September 2020 at the Shirley Ryan AbilityLab in Chicago, IL and Walter Reed National Military Medical Center in Bethesda, MD. The method of recruitment was via physician or prosthetist referral (via phone call or outpatient appointment) and study advertisements on the Shirley Ryan AbilityLab website and social media accounts. Inclusion criteria were: age 18–95, unilateral amputation below the elbow, and demonstrated the ability to use a myoelectric prosthesis. Exclusion criteria were: inability to use a prosthesis, cognitive impairments that would interfere with their understanding of study requirements, or any significant co-morbidity that would preclude completion of the study. The study (ClinicalTrials.gov Identifier: NCT02349035) and online recruitment materials were approved by the Institutional Review Boards of Northwestern University (STU00101444) and Walter Reed National Military Medical Center (IRBNet#410731). All subjects provided written informed consent. The authors confirm that all ongoing and related trials for this intervention are registered.

Subjects were fitted by a certified and licensed prosthetist with a custom-made myoelectric prosthesis consisting of a multi-articulating hand, the i-limb Ultra Revolution (OSSUR [31]), a passive wrist rotation component, and a COMPLETE CONTROL Gen 1 system (Coapt, LLC [32]). Eight pairs of stainless steel dome electrodes were embedded into a flexible inner liner. Two electrodes pairs were placed over the intended sites for the TMR surgery identified with the help of the surgeon and six electrode pairs were placed over additional residual limb forearm muscles. The intention was to take additional care in placing the electrodes in this desired manner, ensuring data from the TMR regions where included in both pre- and post-op TMR surgery prosthetic sockets. Since this was a non-randomized study, pre- and post-surgery home trials were 8-weeks in duration, chosen to be long enough for subjects to become familiar and competent with the prosthesis. All study personnel were non-blinded to participants’ surgical status.

The clinically available pattern recognition system [21, 33], customized to record usage, allowed users to recalibrate their control at any time by making natural muscle contractions that followed along with a series of pre-programmed grips [34, 35]. The speed of hand open and close was proportional to the residual limb muscle activity [36]. To change grips users a sustained a ‘hand open’ muscle contraction signal returning the hand to a natural baseline hand position before performing the natural muscle contraction for the new desired grip. This scheme balanced the potential desire to remain in a grip even if the hand was fully open and only switch to a new grip following a sustained hold open.

### Pre-TMR home trial

Individuals participated in therapy with a certified and licensed occupational therapist to learn how to control the multi-articulating hand with pattern recognition. Clinical judgment and user feedback were used to determine the maximum number of grips the user could access. This determination included a discussion of the user’s activities of daily living, demonstration of the 8 available i-limb grips (a subset of all grips clinically available on the i-limb hand [31]), and practice with the hand with pattern recognition control. The selection of 8 grips included lateral, power, thumb precision pinch opened and closed, thumb 3 jaw chuck opened and closed, standard 3 jaw chuck closed, and index point. No alternative methods of changing grips (e.g., grip chips, IMU-based gesture control, smartphone application, etc) were allowed.

Individuals received a minimum of four training sessions of 2–3 hours in length prior to taking the prosthesis home for at least 8-weeks (Pre-TMR). While at home, users had the ability to use the auto-calibration procedure whenever they desired. During auto-calibration users are prompted to follow along and perform muscle contractions in sync with pre-programmed motions of the prosthesis while EMG data is recorded and auto-labeled for each trained grip. Participants were required to check-in regularly with the occupational therapist, maintain an average usage of at least 2 hours per day with the study device, and log the activities they were performing with their prosthesis. Notably, subjects were allowed to use their own prescribed (i.e., non-study) prostheses if they desired, so long as they maintained an average usage of at least 2 hours per day with the study device. This decision was made as their non-study device may have had a powered wrist rotator or different terminal device (e.g., hook) which may have been more suitable for their occupation and/or some of their activities of daily living. Electronic usage data (duration of time the prosthesis was turned on, which grips were used, and the number of times the prosthesis was recalibrated) was recorded on the pattern recognition controller.

At the conclusion of the home trial, participants returned to the lab to perform a series of outcome measures [37] including: the Southampton Hand Assessment Procedure (SHAP) [38], the Jebsen-Taylor Hand Function Test [39], the Assessment of Capacity for Myoelectric Control (ACMC) [40, 41], the Modified Box and Block Test of Manual Dexterity [42], and the Activities Measure for Upper Limb Amputees (AM-ULA) [43]. A higher score for the SHAP, ACMC, AM-ULA, and Box and Blocks and a lower score for the Jebsen-Taylor indicate better function and/or control. This suite of assessments was used to gain a more representative picture of how differences in grip control effected prosthesis control. Electronic usage data logged on the controller was also downloaded during this time.

### TMR surgery and post-TMR home trial

All participants then underwent TMR surgery in their residual forearm from a board-certified plastic surgeon either at Shirley Ryan AbilityLab or Walter Reed National Military Medical Center. The planned surgery involved transferring the remaining ulnar nerve to the flexor carpi ulnaris muscle and the remaining median nerve to either the flexor digitorum superficialis or brachioradialis muscle [28–30]. Targeted sensory reinnervation [24] was discussed with each individual prior to surgery and optionally performed for those subjects who consented to this additional procedure.

After surgery, participants were allowed to resume prosthesis use of their own prescribed prosthesis as soon as they were comfortable. After at least six months post-surgery, in order to allow the TMR sites to re-innervate and mature, a prosthetist re-checked socket fit of the study prosthesis. Participants received additional occupational therapy to re-assess their selected grips and practice pattern recognition control. They participated in another 8-week home trial (Post-TMR-1) and in-lab outcome measure testing similar to the pre-TMR home trial. Participants then completed one last home trial, this time for 3 months in duration with no minimum usage requirement. They returned to the lab a final time to perform outcome measures (Post-TMR-2). Following fulfillment of all study requirements, participants were allowed to keep the prosthesis used in this study.

A sample size of 9 participants was calculated based on preliminary data from transhumeral and shoulder disarticulation TMR patients on the Box and Blocks Test. We hypothesized that the study’s primary outcome measure, Box and Blocks, and secondary outcome measures (SHAP, Jebson-Taylor Hand Function, ACMC, AM-ULA) will improve following transradial TMR compared to pre-surgery. To compare outcomes measures between pre- and post-TMR surgery, we report values as across-user average changes between pre- and post-TMR surgery time points (i.e., pre-TMR, post-TMR-1, post-TMR-2). From the electronic usage data we calculated average daily wear time as the total number of hours the device was turned on divided by the total number of days the device was turned on. Since the configured grips for each user were selected based on the most functional grips for their own activities of daily living, the time spent in each grip was ranked in order of descending usage and the percentage of time spent in each grip was calculated.

### 32-channel EMG data collection

To further investigate the potential for advanced prosthetic grasp and digit control, a separate EMG data collection was performed following each 8-week home trial pre- and post-TMR surgery was conducted. An array of 32 surface bipolar pre-gelled silver/silver chloride EMG electrodes were placed on each subjects’ residual forearm. Individuals were asked to perform various hand muscle contractions including hand open, 9 different grasp patterns (lateral, power, pinch with the remaining fingers opened and closed, 3 jaw chuck with the remaining fingers opened and closed, index point, hook, and tool), 8 different individual finger and thumb movements (thumb flexion/extension, thumb abduction/adduction, index flexion, middle flexion, ring flexion, and pinky flexion), and rest. Muscle contractions were held for 2 seconds and repeated 8 times for a total of 16 seconds per movement.

An offline analysis was performed on these data using a similar pattern recognition system to what was used during home trial. The data were separated into two separate sets of movements: 1) hand open, 9 different grasp patterns, and rest and 2) 8 different individual finger and thumb movements and rest. For each user and each set of movements, a search was performed to find the optimal 4 channels using classification error (i.e., percentage of incorrectly classified movements divided by the total number of classified movements) as the optimization metric. In this offline analysis, we chose to optimize our analysis using four channels instead of eight channels (which was the total number in the home trial) because clinical experience has found that it is sometimes difficult to place the 16 dome electrodes that are necessary for 8 bipolar EMG channels in a transradial socket. Four channels (and therefore eight dome electrodes) represents a more clinically viable transradial system. Using the 4 optimal channels per user per movement set, a search was performed to evaluate reduction in classification error as additional grips were added to the system. This analysis was performed separately on the pre- and post-TMR data sets.

## Results

**Fig 1:**
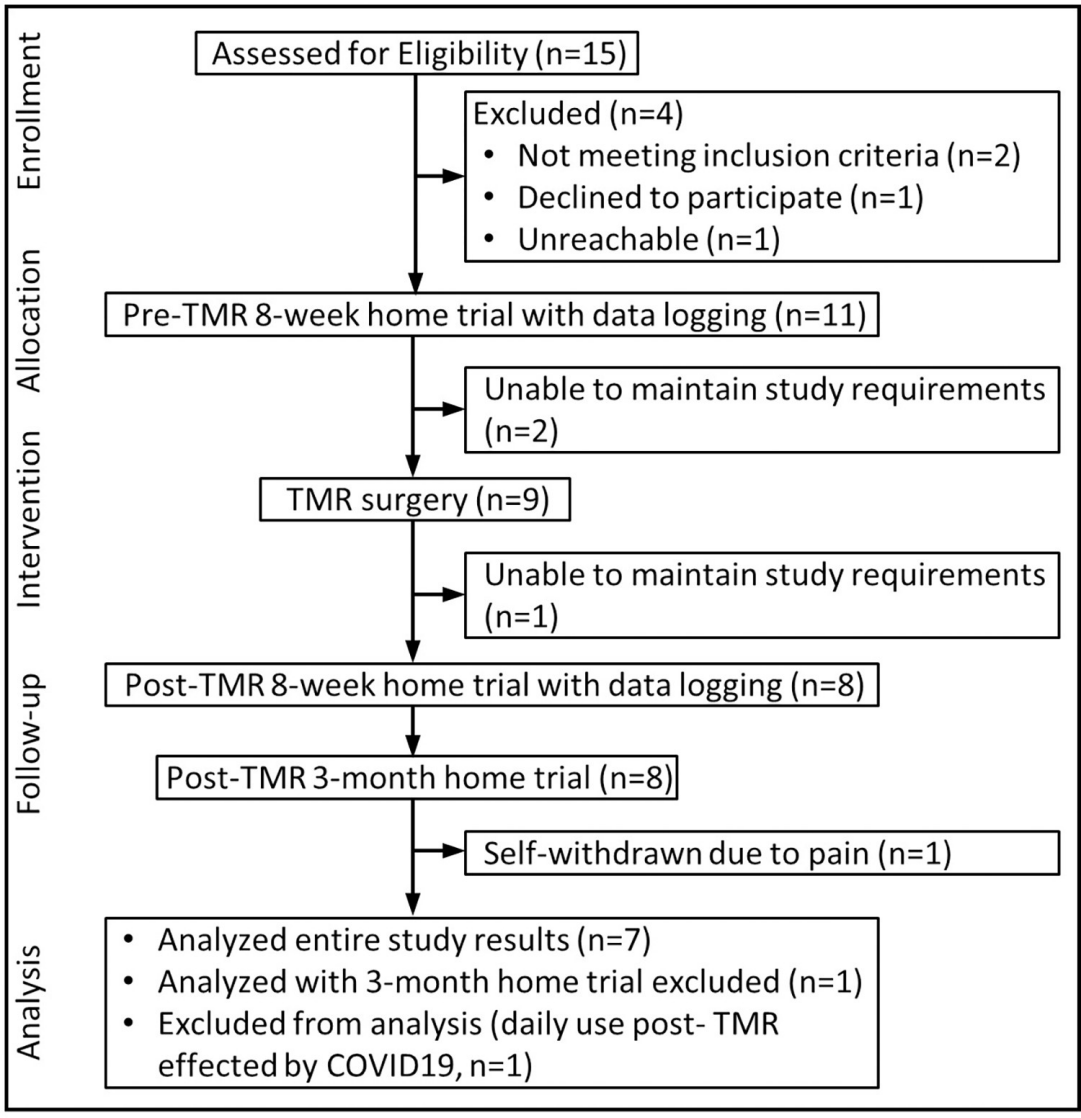
Participant flow diagram of enrollment, allocation, follow-up, and analysis.

Eleven individuals with a unilateral transradial amputation were enrolled in this study (Fig 1, Table 1).

**Table 1:** Participant demographics.

Subject	Gender	Years Post Amputation	Age	Etiology	Prescribed Myoelectric Prosthesis		Study Participation	
					Terminal Device	Myoelectric Control	Home Trials	32-Chan Analysis
TR1	Male	0.8	42	Left Trauma	Sensor speed hand, Motion Control ETD	Direct Control	Yes+	Yes
TR2	Male	1	31	Right Trauma	Michelangelo Hand	Direct Control	Yes	Yes
TR3	Male	1	58	Right Trauma	Motion Control ETD	Direct Control	Yes	Yes
TR4	Male	1.5	29	Left Trauma	Bebionic Hand	Coapt Pattern Recognition	Yes	Yes
TR5	Male	1.5	29	Left Trauma	Bebionic Hand	Direct Control	Yes*	Yes*
TR6	Male	1.5	36	Left Trauma	OSSUR i-Limb,	Coapt Pattern Recognition	Withdrawn	
TR7	Male	2.9	39	Left Trauma	Bebionic Hand, Motion Control ETD	Direct Control	Yes	Yes
TR8	Male	3.4	40	Left Trauma	Bebionic Hand, Motion Control ETD	Direct Control	Withdrawn	
TR9	Male	11	36	Left Trauma	Bebionic Hand, Ottobock Greifer, Motion Control ETD, Sensor speed hand	Direct Control	Withdrawn	
TR10	Female	12	48	Right Trauma	Bebionic Hand, Motion Control ETD	Coapt Pattern Recognition	Yes	Yes
TR11	Male	12.8	53	Right Trauma	OSSUR i-Limb, Sensor speed hand	Direct Control	Yes	No

ETD: electric terminal device

+ TR1 did not complete the Post-TMR-2 home trial

* TR5’s data were excluded from quantitative analysis due to extensive delay in post-TMR testing as a result of the COVID-19 pandemic.

Two subjects withdrew prior to having TMR surgery (TR6 was not able to meet the daily usage requirement and TR8 was unreachable during his second home trial and did not return for outcomes testing) and one subject (TR9) withdrew after TMR surgery prior to the start of the post-TMR 8-week home trial. Seven subjects completed the full study protocol and one subject (TR1) completed all but the final Post-TMR-2 home trial. Of the eight subjects that completed both pre- and post-TMR home trials, one subject (TR5) had his post-TMR home trial during the spring of 2020 which was affected by the COVID-19 pandemic; his post-TMR home usage was severely impacted and his outcomes testing was delayed by 5 weeks. He had not achieved the minimum usage requirement nor used the study prosthesis at all during this 5-week delay. Therefore, his data were excluded from the quantitative analyses.

### Prosthesis control and usage

**Fig 2:**
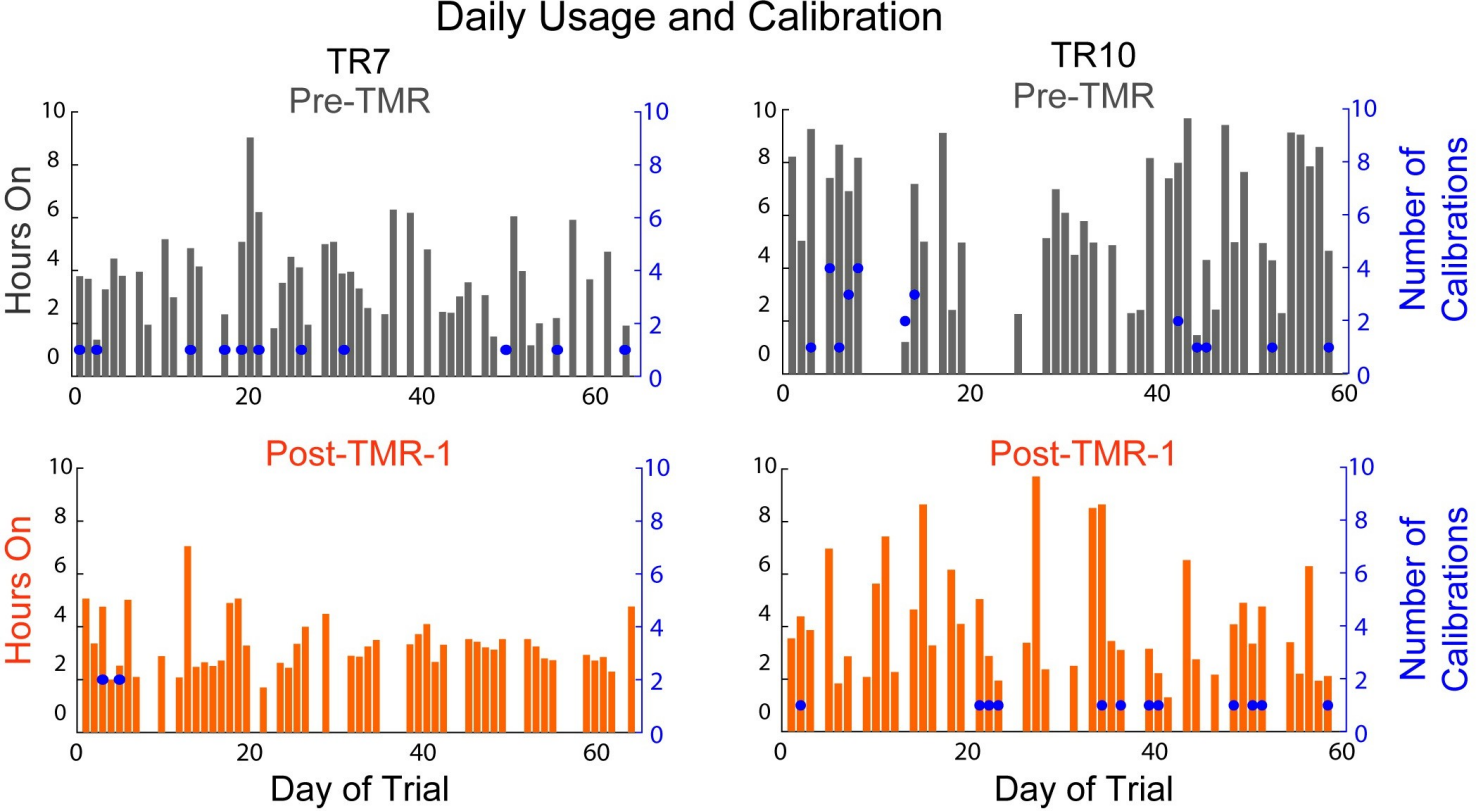
Home usage from two representative participants during the 8-week Pre-TMR (*top*) and 8-week Post-TMR-1 (*bottom*) home trials. Each graph indicates the number of hours the device was turned on (bar, with scale on left-hand axis) and the number of calibrations (dot, with scale on right-hand axis) performed for each day of the home trial. Days of no use were logged as 0 hours on the plots.

All users successfully used the multi-articulating hand prosthesis both before and after TMR surgery. Only minor fitting adjustments were necessary to the socket post-surgery; the location of all the electrodes remained the same for all subjects. Comparing between the pre-TMR and post-TMR 8 week home trials, there were no significant differences between the number of configured grips, the cumulative hours powered on, nor the number of recalibration sessions (Table 2) (Fig 2). Grip usage was similar between surgery conditions: on average pre-TMR individuals spent 56.1% [6.6] of the time spent in the preferred grip, and 23.4% [3.5] and 16.5% [3.4] of time spent in the 2nd and 3rd most used grips. Post-TMR individuals spent 52.5% [7.2] of the time spent in the preferred grip, and 24.1% [3.6] and 13.6% [2.6] of time spent in the 2nd and 3rd most used grips.

**Table 2:** Pre- vs post-TMR 8-week home trial usage data.

Metric		Pre-TMR	Post-TMR-1	Post-TMR-2
Number of grips configured		3.7 [0.8]	4.1 [0.7]	4.1 [0.7]
Home trial length in days		69.7 [8.9]	72.6 [10.5]	119.3 [33.0]
Number of days turned on		51.1 [15.7]	43.4 [18.2]	20.8 [11.3]
Cumulative hours on		264.6 [67.1]	211.4 [57.1]	67.8 [38.4]
Number of calibrations		33.1 [30.6]	12.6 [11.1]	11.2 [7.1]
Grip usage as a percent of total time in hand close				
	Most used grip	54.0 [19.0]	46.0 [9.5]	42.4 [11.2]
	2^nd^ most used grip	23.8 [10.7]	27.4 [5.0]	27.3 [5.1]
	3^rd^ most used grip	18.1 [9.2]	15.5 [5.7]	18.8 [5.3]

Values represent mean [standard deviation]

**Fig 3:**
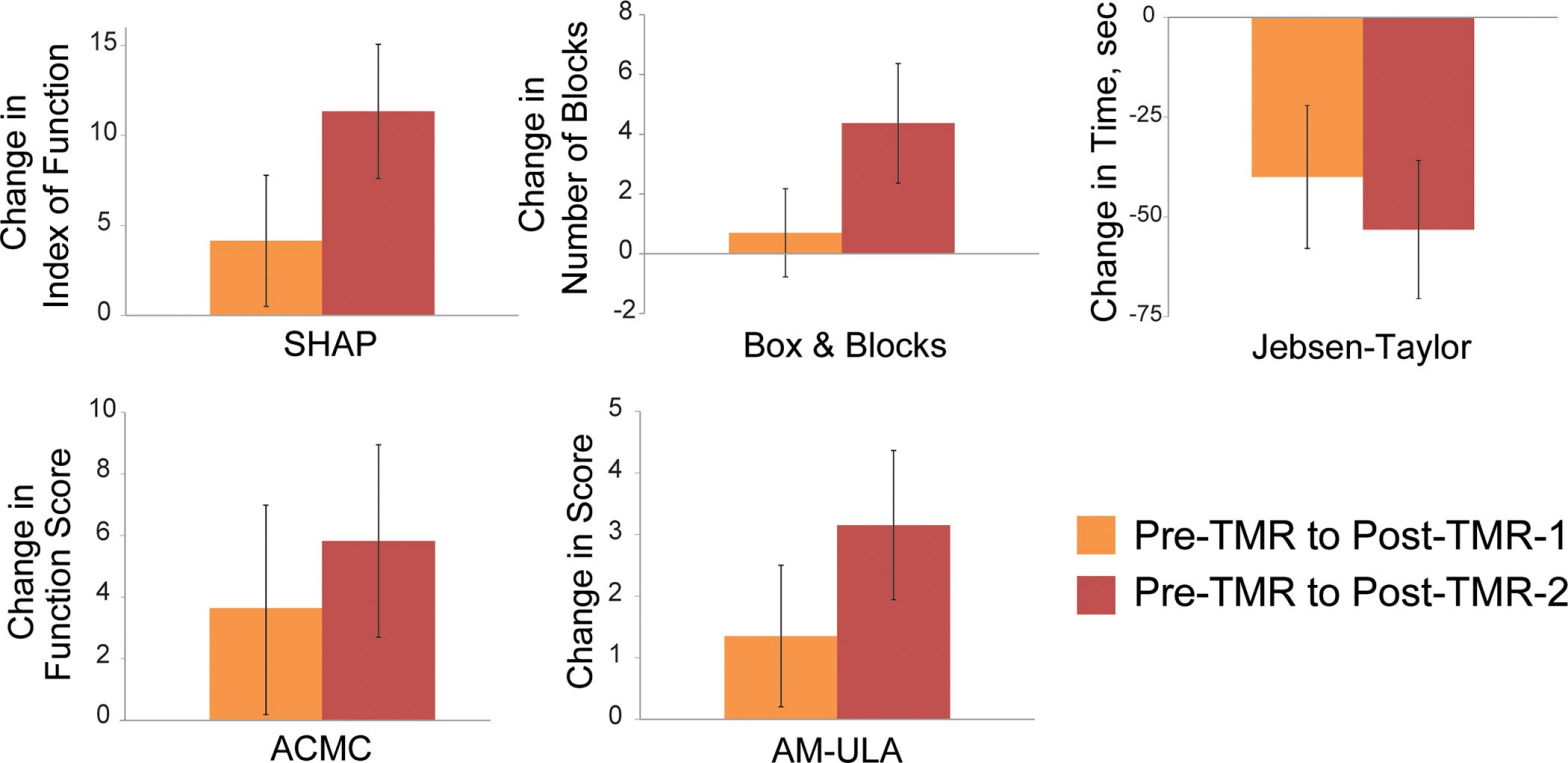
Changes in post-home trial outcome measures for 7 subjects from the Pre-TMR 8-week trial to the Post-TMR 8-week trial (Post-TMR-1, *orange*) and from the Pre-TMR 8-week trial the final Post-TMR 3 month trial (Post-TMR-2, *red*). The SHAP, Box & Blocks, and Jebsen-Taylor showed the largest improvements Post-TMR-2 compared to Pre-TMR.

Fig 3 shows the overall average change between pre- and post-TMR data points for each outcome measure. Users’ index of function score on the SHAP showed an average improvement of 12.6 points following the final 3-month post-TMR home trial (Post-TMR-2) compared to pre-TMR (Fig 3, Table 3). Subjects completed testing in a shorter time on the Jebsen-Taylor test of hand function (i.e., average decrease of 52.2 seconds) during the Post-TMR-2 condition compared to the Pre-TMR condition. Users transferred more blocks, on average 5.8 more blocks, during the Post-TMR-2 Box and Blocks test compared to Pre-TMR. No differences were found between TMR condition for the ACMC or AM-ULA.

**Table 3:** Outcome measures following home trial.

Outcome	Pre-TMR	Post-TMR-1	Post-TMR-2
SHAP, index of function	35.9 [2.6]	40.0 [5.1]	48.0 [4.3]
Jebsen-Taylor, seconds	225.1 [19.0]	185.1 [16.9]	174.2 [10.4]
ACMC, score	59.3 [2.2]	62.8 [2.7]	65.9 [3.3]
AM-ULA, score	21.0 [1.1]	22.3 [0.9]	23.5 [0.9]
Box and Blocks, number of blocks	18.8 [1.6]	19.5 [1.0]	23.7 [1.0]

Values represent mean [standard deviation]

**Fig 4:**
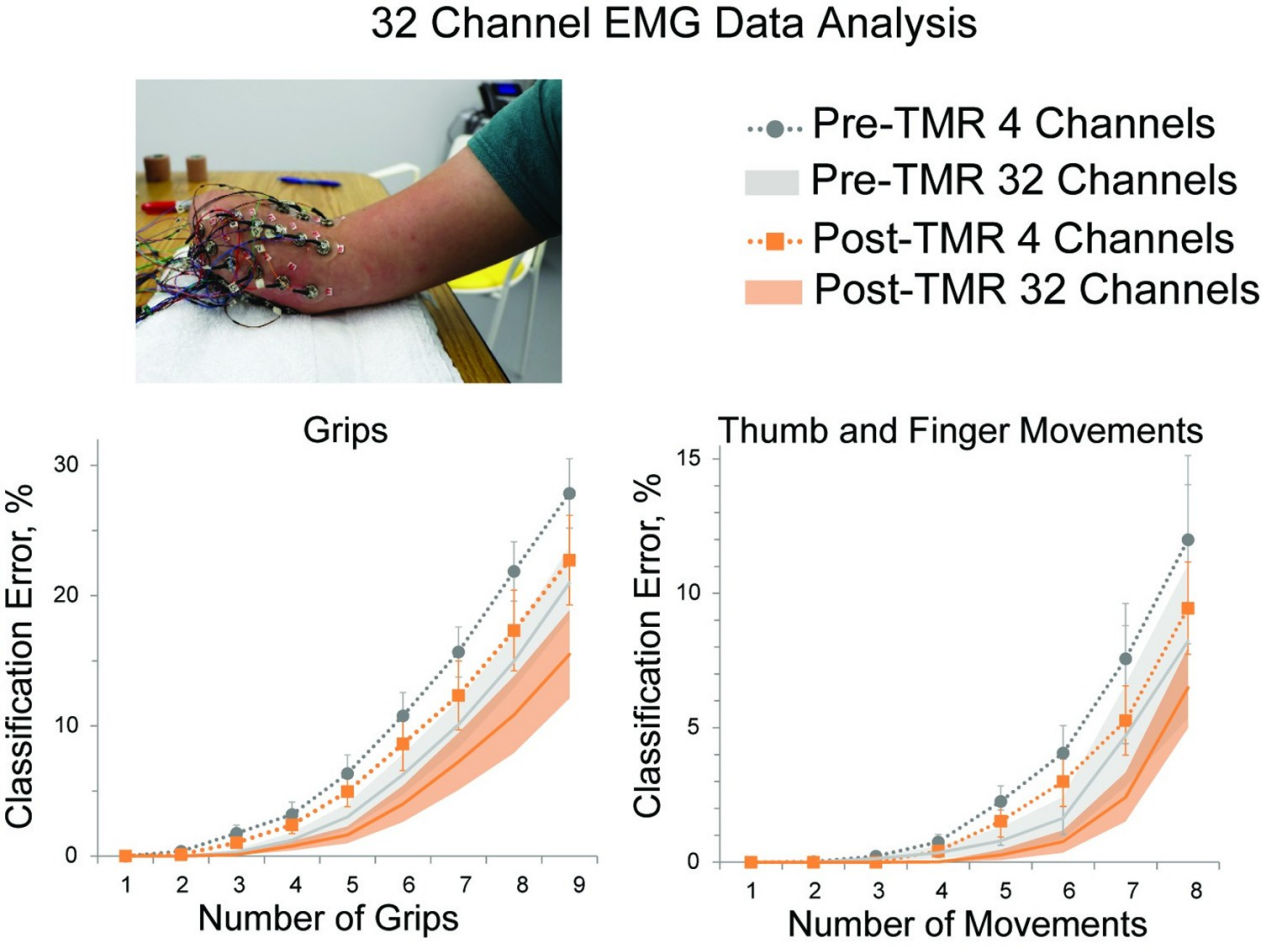
32-channel EMG data collection setup (*top*) and results (*bottom*) for 6 subjects. Offline classification error rates are plotted for the system with increasing number of grips configured (*left*) and thumb and finger movements (*right*). Colors represent data collected Pre-TMR (*grey*) and Post-TMR (*orange*) optimized for a system using only 4 channels and all 32 channels. Standard deviations for the 4 channels are shown with error bars and for the 32 channel data with shaded bands.

Offline classification errors from the 32-channel EMG data collection using all 32 channels as well as the 4 optimal channels show a slight reduction in error rate Post-TMR compared to Pre-TMR for the grip movement set as well as the individual thumb and finger movement set (Fig 4). When only 4 grips were configured, similar to the amount configured during the home trial portion of this study, classification error rate was 3.2% [0.1] for the 4-channel Pre-TMR data and 2.4% [0.7] for the Post-TMR data. For all 9 grips, error rate was 27.8% [2.7] for the 4-channel Pre-TMR data and 22.7% [3.4] for the 4-channel Post-TMR data.

### TMR and TSR qualitative results

**Fig 5:**
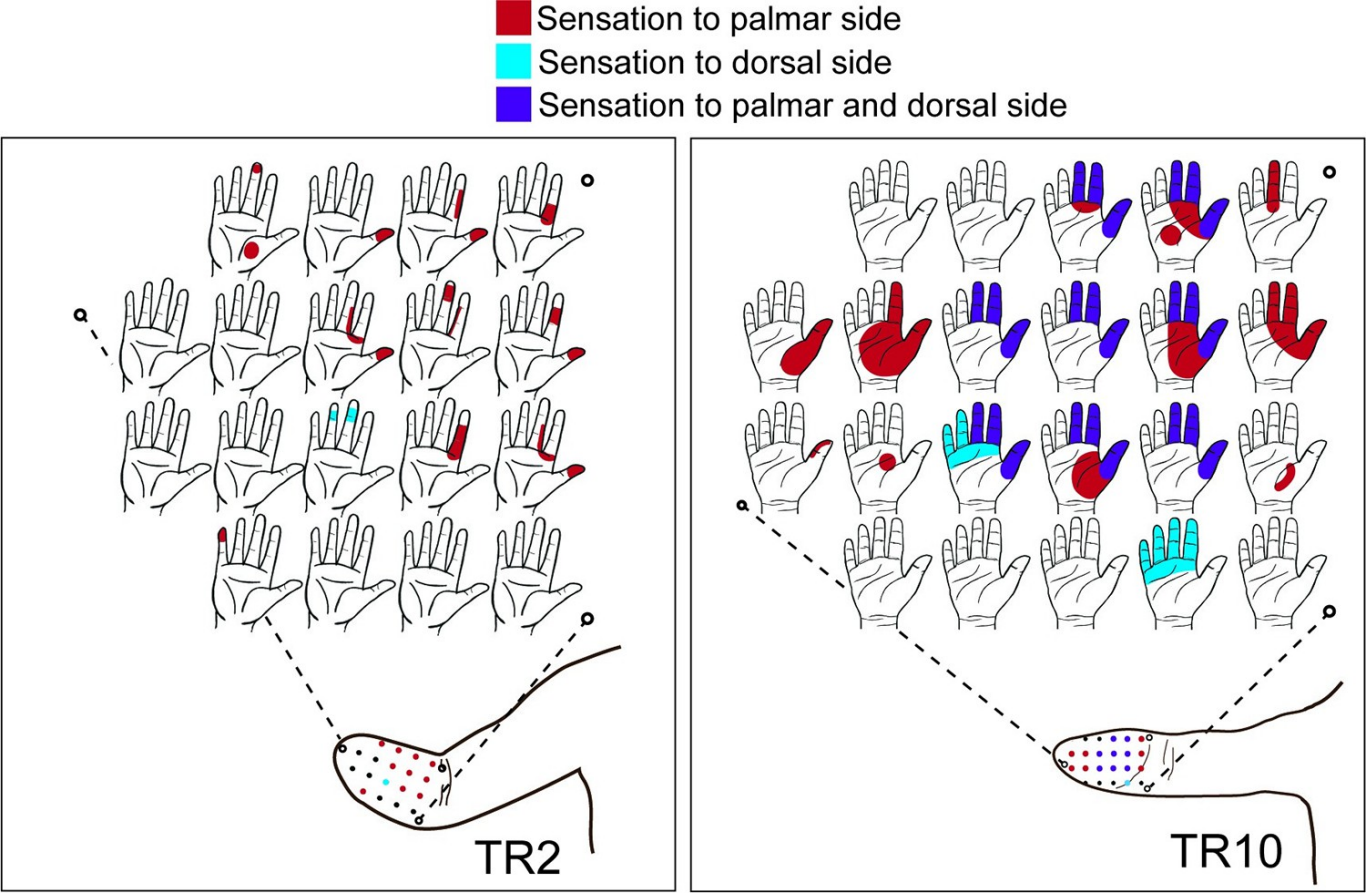
Reinnervated forearm skin of the two subjects who underwent targeted sensory reinnervation. For both individuals, sensations referred from their missing hand were felt in their residual forearm. Referred sensations were localized to either the palmar side (red), the dorsal side (blue), or both (purple).

Table 4 summarizes the TMR surgeries for the eight subjects who completed the protocol; two subjects opted to also have TSR. Four subjects noticed an improvement in their phantom limb resting position and/or movement of their phantom hand at the six-month mark or later following TMR surgery. Six subjects (TR2, TR4, TR5, TR7, TR10, TR11) indicated a reduction in overall self-reported pain levels and/or an ability to reduce their overall medications for pain management post-TMR. One subjects (TR3) had no noticeable self-reported change in pain levels and one subject (TR1) who had an increase in residual limb pain post-TMR surgery underwent an additional surgery by a different surgical team after completion of the 8-week post-TMR home trial for neuroma revision of a different nerve. This subject did not complete the Post-TMR-2, 3 month home trial. Four subjects indicated positive improvements post-TMR in the resting position or movement of their phantom limb (Table 4). The two subjects who underwent TSR surgery developed referred sensations from their missing hand in their residual forearm (Fig 5).

**Table 4:** Subject TMR and TSR results.

Subject	TMR nerve transfers		TSR	Phantom Limb Position	
	Median	Ulnar		Pre-TMR	Post-TMR
TR1	Brachioradialis	FCU	none	Hand feels “trapped”, minimal ability to move fingers	No noticeable change
TR2	Brachioradialis	FCU	Median sensory nerve to distal aspect of lateral antebrachial cutaneous nerve	Hand feels as if fingers are “wrapped around each other”, cannot move fingers	Fingers rest in a more relaxed position and able to move thumb and fingers slightly
TR3	Brachioradialis	FCU	none	Hand feels mostly “bound up” in a closed fist but sometimes feels “half open”	Resting position remains the same but now can feel minimal thumb and finger movements
TR4	FDS	FCU	none	Hand in constant grip	Able to move thumb and fingers
TR5	FDS	FCU	none	Hand remains in half open fist with minimal ability to move fingers (not thumb)	Resting position remains the same but now pointer and middle finger move together
TR7	Brachioradialis	FCU	none	Able to move thumb and fingers	No change
TR10	Brachioradialis	FCU	Median sensory nerve to end of lateral antebrachial cutaneous nerve	Hand remains in half closed fist, ability to wiggle fingertips	Hand position slightly more open and able to move thumb and fingers more
TR11	FDS	FDP	none	Hand remains in semi-closed fist, very difficult to open fingers	No noticeable change

FDS: digitorum superficialis; FCU: flexor carpi ulnaris; FCR: Flexor carpi radialis; FDP: flexor digitorum profundus

## Discussion

For the participants with a transradial amputation included in this study, our results demonstrate some benefits of TMR surgery. Outcomes data indicate that control improvements were seen following the Post-TMR-2 home trial compared to the Pre-TMR home trial for the SHAP, Jebsen-Taylor and Box and Blocks. The SHAP, a timed outcome measure that evaluates unilateral hand functions required for activities of daily living [30], improved by 12.6 points. This increase may indicate that these users had an improved ability to function with the prosthesis post-TMR; it is also possible that it is from users gaining a better understanding of the prosthesis or from learning the assessment [44]. Users decreased their timed score on the Jebsen-Taylor by an average of 52.2 seconds during the Post-TMR-2 outcomes testing compared to Pre-TMR. The Box and Blocks improvement of 5.8 more blocks is below the minimal clinically important difference at the 90% confidence level of 6.5 blocks [45]. With the limited numbers available, potential differences in the ACMC and AM-ULA were not different between assessment points.

The Post-TMR-2 home trial occurred approximately 9–12 months post-TMR surgery. It is likely that the targeted muscles continued to reinnervate and strengthen over the year following the TMR surgery, which is our primary explanation for the improved outcomes; however, there are other potential explanations. Control improvements may have been due to users having more experience with the multi-articulating hand prosthesis. Due to the nature of the study protocol of users serving as their own baseline for TMR surgery, we were unable to randomize the condition order: 8-week Pre-TMR home trial, 8-week Post-TMR-1 home trial, and 3 month Post-TMR-2 home trial. While additional time with the prosthesis may allow users to better predict hand speed and how the hand moves within each grasp pattern it is important to note that the first two home trials constituted an average of 51 and 48 usage days, providing the users with experience using the prosthesis. It is also possible the users simply learned to complete the tests better. However, given the length of time between the tests, we feel this contribution would be small if it existed at all. Individuals with a transradial amputation have shown to improve their pattern recognition-based control of a virtual prosthesis over the course of 10 days of training [46]. Additionally, transradial users were shown to improve on pre- and post- home test outcome measures after a month of pattern recognition home use [47]. In this same study, users did not show similar gains pre- and post-home trial for conventional, direct control, indicating that learning of the prosthesis (e.g., hand speed, how it grasps, etc) likely occurs on a shorter timeline than learning pattern recognition control. Although are not yet studies to indicate the timeline of if/when pattern recognition learning is completed, there are differences in EMG patterns created by novice and experienced users [48]. It is also possible that some of the qualitative TMR surgery outcomes aided users’ consistency and control post-TMR surgery. Reductions in pain, changes in phantom limb sensations, and improved phantom limb awareness may have led to increased repeatability of the muscle contractions necessary for pattern recognition prosthetic control. Further analysis of the calibration data may shed light on whether changes in phantom limb representations and/or long-term pattern recognition use can lead to increased repeatability or separability of EMG signals [48].

The 32-channel EMG analysis also showed a decrease in classification error post-TMR surgery compared to pre-TMR surgery, although these differences were more prominent with a more complex system (i.e., when all 9 grips or all 8 thumb and finger movements were included). Additionally, this analysis showed that four optimally placed EMG channels could provide a highly accurate pattern recognition system: under 10% error for 5 grips pre-TMR or 6 grips Post-TMR and under 20% error for 7 grips pre-TMR or 8 grips post-TMR (Fig 4). Similar findings with individuals with a transradial amputation for channel optimization have been reported: Li et al. [16], found only six optimally placed electrodes resulted in 8.5% error for 10 movements of a virtual transradial prosthesis and Daley et al. [49], found accuracy remained relatively high for transradial amputees using four electrodes. While likely an optimal number of channels can be found, caution should be taken interpreting these results since these analyses have only been performed offline. Additionally, for the analysis in this EMG analysis, grip patterns were chosen based on which ones were most separable from others in the set as opposed to choosing grips based on what may be most functionally relevant to an individual’s activities of daily living. Therefore, in clinical practice the likelihood is that these systems (or systems that use up to 8 channels) will create slightly higher error rates in grip selection.

The current study saw a smaller improvement in functional prosthesis control than previous studies investigating TMR surgery following more proximal upper limb amputation. This is likely because TMR surgery subsequent to more proximal level amputation allows for access of hand and wrist information where there was none prior. Conversely, following transradial amputation there already exists hand and wrist neural information due to native innervation of the forearm and extrinsic hand muscles. For the subjects in this study, activation of the newly reinnervated muscles either may not have added considerable neural content or these TMR signals were small and overshadowed by surrounding forearm activation for similar hand movements. It is important to note that denervation of the target muscles used in this study (flexor carpi ulnaris, flexor digitorum superficialis, and brachioradialis muscles) did not lead to worse prosthetic control nor negatively affect residual limb control.

Four subjects described improvements to either the resting position of their phantom hand or the amount of movement they are able to visualize with their phantom. For example, one subject described the resting position of his phantom limb as fingers tightly clenched over one another with a feeling that his fingernails were going through his palm. It had remained that way since his amputation. Post-TMR his phantom hand relaxed such that his hand was more open and in a more relaxed hand position. Additionally, six subjects saw reductions in pain and/or were able to reduce their pain medication levels post-TMR surgery. The one subject who reported no change in pain, however, was no longer taking pain medication that he had been pre-TMR. A recent TMR clinical trial on treatment of neuromas [26] found significant decreases in phantom limb pain and a trend towards reduction in residual limb pain post-TMR surgery compared to conventional treatment of neuromas. One subject who did not experience a reduction in pain, was found to have additional symptomatic neuromas post-TMR surgery due to additional amputated nerve endings that were not targets of the TMR surgery. Evidence shows that post-TMR, the newly cut motor nerves do not typically become symptomatic neuromas [25, 26, 50].

The results of this study may not be fully generalizable to the full population due to some limitations of the study design. Recruitment for this study was challenging due subject consent and retention for the year-long study testing protocol as well as the surgical component. Additionally, because the intervention was TMR surgery, the order of conditions could not be randomized. Therefore the results may be prone to biases including selection bias and regression to the mean. And since there was no control group (i.e., group of individuals with a transradial amputation whom were tested at the same intervals without undergoing TMR surgery) it is not possible to quantify the potential for additional learning, if any, to have occurred during the post-TMR conditions. Ultimately, the number of subjects who enrolled and completed the study was lower than planned for due to unexpected subject dropout and the impact of the COVID-19 pandemic. Due to these potential biases, care should be taken while interpreting our results; as such statistical analyses were not included to avoid over generalizing the results.

The current study also had other limitations. Because the specific goal was to investigate potential improvements in control due to TMR surgery and the transferred nerves provided additional hand control information, powered wrist control was not included. It is unclear if the addition of a powered wrist would have impacted control or functional outcomes. All participants continued to wear their own, clinically prescribed prosthesis during the home trials; this was allowed as long as they were able to maintain the minimum average usage of 2 hours per day with the study prosthesis. We cannot say definitively what the total prosthesis wear-time (study and non-study devices) was for users. Subjects chose to use their clinically prescribed devices for a variety of reasons including the addition of a powered wrist rotator or the preference to use a different terminal device.

## Conclusion

Transradial amputation is the most common arm amputation and although affected individuals can control wrist and hand movements of a powered prosthesis, functional and reliable control still remains a challenge. The goal of this study was to determine if these individuals could benefit from the additional neural information that TMR surgery provides. Our results show that the subjects included in this study showed functional control benefits of using a multi-articulating hand prosthesis 9–12 months post-TMR surgery. Additional qualitative TMR and TSR benefits were observed in some, but not all, enrolled subjects. Future work incorporating both the sensory feedback benefits for individuals with transradial amputation as well as the potential for TMR surgery to result in more repeatable muscle contractions, possibly due to the reduction in pain levels and/or changes to phantom limb sensations, has the potential to increase functional use of many of the clinically available dexterous prosthetic hands.

## Supporting information

S1 Checklist TREND statement checklist. (PDF)

S1 File (PDF)

S1 Data (XLSX)

S2 Data (XLSX)
