# Supplementary material for: Myoelectric prosthesis hand grasp control following targeted muscle reinnervation in individuals with transradial amputation
Source: PLoS One. 2023 Jan 26;18(1):e0280210. doi: 10.1371/journal.pone.0280210 (PMC9879512; doi:10.1371/journal.pone.0280210)
Supplement: S1 File — (PDF) [file pone.0280210.s002.pdf]

Application of Targeted Reinnervation for People with Below Elbow Amputation

PRINCIPAL INVESTIGATOR:	Name	Todd Kuiken, MD, PhD
	Address	Shirley Ryan AbilityLab<br>355 E. Erie St., 11th floor<br>Chicago, Illinois 60611
	Phone #	312-238-2080
	Fax #	312-238-2081
	Email	tkuiken@northwestern.edu
PARTICIPATING SITES:	Name(s)	Gregory Dumanian, MD
	Institution(s)	Northwestern University FSM
	Phone #	312-926-6022
	Email	gdumania@nmh.org
	Name(s)	B. Kyle Potter, MD
	Institution(s)	Walter Reed National Military Medical Center
	Phone #	301-400-2727
	Email	Benjamin.K.Potter.mil@health.mil
	Name(s)	Mickey Cho, MD
	Institution(s)	San Antonio Military Medical Center
	Phone #	210-916-5666
	Email	mickey.cho@us.army.mil

FUNDING AGENCY: National Institutes of Health and Center for Bionic Medicine Philanthropy

Synopsis

Title	Application of Targeted Reinnervation for People with Transradial Amputation
Short Title	Transradial TMR
Protocol Date	August 1, 2019
Study Duration	5 years
Study Center(s)	Shirley Ryan AbilityLab, Northwestern University, Walter Reed National Military Medical Center, San Antonio Military Medical Center
Objectives	*Aim 1.* Quantify and compare conventional and pattern recognition control of the highly articulated below elbow prostheses with a multifunction hand capable of many hand grasps and postures.<br>*Aim 2.* Perform TMR surgery and evaluate below elbow TMR pattern recognition control of multifunctional prosthesis.<br>*Aim 3.*Evaluate the information content of reinnervated muscles following below elbow TMR.
Number of Subjects	16
Diagnosis and Main Inclusion Criteria	16 Below Elbow Amputees

TABLE OF CONTENTS

(May be revised and numbered as necessary, but should include major sections)

Synposis..

1.0 Introduction..

2.0 Study Objectives..

3.0 Selection of Subjects..
3.1 Inclusion Criteria..
3.2 Exclusion Criteria..

4.0 Subject Registration..
4.1 Informed Consent Process..

5.0 Study Design & Methods..
5.1 Experimental Protocol..

6.0 Statistical Plan..

7.0 Safety Assessment..
7.1 Risk and Injury..
7.2 Data Collection & Record Keeping..

8.0 Benefits..

9.0 Compensation..

10.0 References..

Appendices..

1.0 INTRODUCTION - BACKGROUND AND RATIONALE

An estimated 41,000 persons in the US live with major upper limb amputation (at the wrist level or higher) [5], including over 200 service men and women with major upper limb amputations resulting from military operations in Iraq and Afghanistan [6]. Transradial amputation is the most common level of major upper limb amputation, accounting for over 40% of major upper limb amputations in the general public [1], and nearly 50% in military personnel over the last decade [6]. Between 1988 and 1996 (the last period for which statistics are available), there were 5,839 recorded transradial amputations in the United States [1]. The majority of upper-limb amputees experience amputation due to trauma [7] and have active lifestyles. Expectations for function of upper limb prostheses are high because of the standard established by able-bodied dexterity. However, it is estimated that only 50% of patients with an upper limb amputation use a prosthesis [8]. Of these, less than half use externally powered prostheses. Thus prosthesis use is surprisingly low, due in large part to poor function of the devices [9].

Standard treatment of transradial amputation involves fitting with either simple body-powered or myoelectric prostheses. For body-powered prostheses, harnesses and cables are used to translate body movements (such as shoulder shrugging) into operation of the terminal device. For myoelectric transradial prostheses, electromyographic (EMG) signal amplitudes are recorded from forearm muscles to control only a single degree of freedom (DOF); control of an additional DOF requires awkward switching using muscle co-contraction, operation of mechanical switches, or even a cell phone application. In recent years, an exciting array of new multifunction hands has been released to the market and several other hands and wrists are under development. Many of these new multifunctional hands allow for many functional hand grasps and postures to be used.

Targeted Muscle Reinnervation (TMR) and Pattern Recognition Control: Targeted muscle reinnervation (TMR) is an innovative surgical technique that has led to a significant advancement in the control of multifunction prostheses [13-15]. During TMR surgery, nerves left truncated by amputation are transferred to spare (target) muscle sites and, over several months, reinnervate these muscles. The reinnervated muscles provide additional neural control information, including EMG data representing intrinsic hand muscles [16]. The success rate of TMR in creating new control sites is very high (>94%) [17], this is because relatively small muscle regions are reinnervated by large nerves, providing a vast excess of motor nerve fibers [18]. TMR results in significant functional improvements in myoelectric prosthesis control for proximal-level upper extremity amputees [13-15]. Even when using only conventional two-site control paradigms, these amputees at the CBM-SRALAB have shown marked clinical improvement after TMR. They have demonstrated a 2.5- to 7-fold increase in the speed of task performance [15], and have reported that controlling their prosthesis is significantly easier and more natural [13, 14, 19, 20]. TMR is gaining widespread acceptance and has now been performed on over 75 patients at multiple institutions worldwide.

In myoelectric pattern recognition control, a machine-learning algorithm is first formed with training data [21, 22]. With linear discriminant analysis (LDA), for example, features are extracted that reduce the EMG data while retaining the most important discriminating

information. The LDA classifier then separates the features in multidimensional space and draws boundaries between the different classes of activity. Once trained, the algorithm analyzes subsequent EMG data to determine which class of movement the user is trying to perform. When the user elicits a contraction corresponding to the desired movement, the classifier recognizes the pattern of muscle activity and chooses the appropriate motion. As a result, pattern recognition provides the user with intuitive control and allows rapid selection of multiple prosthesis movements. This eliminates the need for burdensome switching techniques and allows the user to control multiple functions with a small number of recording electrodes. Whereas conventional myoelectric control uses the smoothed EMG amplitude as the only source of information, pattern recognition looks at various features of the recorded EMG signals; this greatly increases control performance [23]. Among the various classification techniques used to implement EMG pattern recognition [23-31], all have demonstrated similar high classification accuracies (92%–98%) [32].

TMR has the potential to restore intrinsic hand muscle data for below elbow amputees. We expect that TMR in below elbow amputees will provide sufficient neural control information to greatly improve pattern recognition control of multifunction prostheses. The successful application of TMR would minimize or eliminate the need for complex switching routines and provide intuitive, efficient operation, thereby revolutionizing the standard of care and resulting function for below elbow amputees. Furthermore, we will expand the science of neural-machine interfaces. In particular, high-density EMG experiments will be a valuable resource for understanding the contribution of each nerve transfer and the resulting data sets will be important for numerous studies on enhancing prosthesis control.

2.0 OBJECTIVES

Aim 1: Quantify and compare conventional and pattern recognition control of the highly articulated below elbow prosthesis with a multifunction hand capable of many hand grasps and postures.

Aim 2: Perform TMR surgery and evaluate below elbow TMR pattern recognition control of multifunctional prostheses.

Aim 3: Aim 3: Evaluate the information content of reinnervated muscles following below elbow TMR.

3.0 SELECTION OF SUBJECTS

3.1 INCLUSION CRITERIA:

- Ages 18-95
- An upper limb amputation below the elbow.

3.2 EXCLUSION CRITERIA

- Significant new injury that would prevent use of a prosthesis: The ability to consistently wear a prosthesis and perform activities of daily living and specific performance tasks is necessary to evaluate the relative benefits of the interventions.

- Cognitive impairment sufficient to adversely affect understanding of or compliance with study requirements, ability to communicate experiences, or ability to give informed consent: The ability to understand and comply with requirements of the study is essential in order for the study to generate useable, reliable data. The ability to obtain relevant user feedback through questionnaires and informal discussion adds significant value to this study.

- Significant other co morbidity: Any other medical issues or injuries that would preclude completion of the study, use of the prostheses, or that would otherwise prevent acquisition of useable data by researchers

4.0 SUBJECT REGISTRATION

We will consult with clinical teams at both civilian hospitals/clinics and with the clinical teams at San Antonio Military Medical Center (SAMMC) in San Antonio, Texas and Walter Reed National Military Medical Center (WRNMMC) in Bethesda, Maryland to help with recruitment of previous patients. Recruitment will also occur through the Shirley Ryan AbilityLab Facebook, Twitter and Instagram accounts with use of the SRALAB website study details including inclusion criteria and contact information.

Each subject will initially be approached by his or her own personal physician, prosthetist, or occupational therapist, so initial discussions will be private and protected. The Chain of Command will not be present during the Soldier’s briefing, consenting, and participation. Assuming that the subject is willing to be contacted by study staff, they will be contacted by phone and/or letter (post or email). Written information explaining the trial procedure, time commitment, and goals of the study will be provided to the subject as well as contact information for study organizers and appropriate patient representatives/advocates from SRALAB or the other institutions. If the potential subject still indicates an interest in participating in the study, he/she will also be sent a copy of the consent form. Ample time will be provided so that the subject can discuss the study with significant others, such as family members, employers, or other members of his or her clinical team. Subjects will be free to decline participation in the study. Inducements (where applicable under federal law) will be limited to reimbursement for time and expenses while involved in the study. Due to federal law, active-duty military personnel will not be compensated for research participation unless they are

off duty or on leave during the time of their participation. Subjects will be recruited without bias to race or gender. This study will be listed on the Shirley Ryan AbilityLab website with contact information for recruitment purposes.

4.1 **Informed Consent Process**

Informed consent to participate in the study will be obtained per IRB protocol. We will send the consent form to the subject prior to the first visit to Shirley Ryan AbilityLab (SRALAB) or WRNMMC SAMMC. We will be available, by phone, to answer any questions about the study prior to the first visit. At the first visit, we will again review the study verbally, including time commitments and potential risks. The subjects will be asked if they still wish to participate and will be given an informed consent form, which they will be asked to sign. A researcher will witness the consent process and be available to answer any questions that arise during the informed consent process. The researcher will also assign a confidential subject code to the participant. Subjects will be informed that participation is strictly voluntary and that they can withdraw from any experiment at any time for any reason without consequence.

5.0 STUDY DESIGN & METHODS

We propose a three-site phase II clinical trial of TMR for below elbow amputees: the sites include the Center for Bionic Medicine at the Shirley Ryan AbilityLab (CBM-SRALAB), Walter Reed National Military Medical Center (WRNMMC), and the San Antonio Military Medical Center (SAMMC) as collaborating sites. The study will be performed at each site, with CBM-SRALAB leading the study and responsible for adequate training of all personnel at all sites.

The overall study is designed with two Specific Aims and three parts: (1) a two-way randomized crossover study to compare conventional and pattern recognition control of advanced multifunction prostheses, and (2) a repeated-measures comparison of pre and post-TMR pattern recognition control of these myoelectric devices.

5.1 Experimental Protocol

Up to 16 below elbow subjects are expected to be enrolled in this study. Eight (8) of the subjects will receive prosthetic fitting and TMR surgery at the Shirley Ryan AbilityLab. Eight (8) subjects will receive prosthetic fitting and TMR surgery at the Walter Reed National Military Medical Center, or the San Antonio Military Medical Center. All prosthetic training and testing will be performed by the Shirley Ryan AbilityLab. Informed consent to participate in the study will be obtained per institutional protocol. Subjects will be told the inclusion and exclusion criteria and asked to provide a yes/no answer as to whether they can participate in the study. After signing the consent form, each subject’s screening inclusion/exclusion criteria will be documented.

Phase 1:

The subjects will travel to one of the centers for their initial recruitment and fitting with the multifunction hand system or a multifunctional hand and wrist system. The prosthesis system will consist of a custom-made socket and commercially available components, including the TASKA hand. During the initial recruitment and fitting visit, subjects will also be seen by one of the surgeons on the study team in order to identify potential TMR sites on their limb.

The subjects will be randomly assigned to begin with conventional control or pattern recognition control. For conventional control, clinical best practice will be used in placing one electrode over the forearm flexors and one over the extensors. For pattern recognition control, a grid of eight electrodes will be used to record the EMG signals: a ring of four electrodes will be located around the proximal upper forearm; and a second ring of eight electrodes will be located more distally on the residual limb. The Coapt complete control system will provide both conventional control paradigms and pattern recognition control. It will also be programmed to quantify the amount of time the prosthesis is turned on, how often the prosthesis is used, and what specific functions are used. This data will be important for the comparative effectiveness analyses.

Conventional Control Testing: Subjects will be trained by an Occupational Therapist and prosthetist in conventional control of the prosthesis. The days of prosthesis training and testing may take place at the Shirley Ryan AbilityLab, Walter Reed National Military Medical Center, or the San Antonio Military Medical Center or near your home. Study coordinators will determine the best location. The prosthetic training days may occur concurrently with the fitting appointment or it may require a separate visit. The subjects will use the prosthesis at home for at least 8 weeks. They will be asked to use the prosthesis for an average of 2 hours a day, and to keep a daily log of how much they use the device. They will also be asked to take notes on the usefulness of the device, and check in weekly with a therapist to briefly discuss the usage and prosthesis performance. After at least 8 weeks, the patients will return to the center for testing or an OT will go to their home. Our full outcomes toolbox will then be used to assess their performance, and usage data from their prosthesis will be downloaded.

Pattern Recognition Control Testing: The occupational therapist will train the patient in using pattern recognition control. We will use our pattern recognition training program, which includes conceptual training, control training, functional use training, and recalibration training [43]. The prostheses will be equipped with prosthesis-guided training to enable the patient flexibility in device training. Subjects will then use their pattern recognition prostheses at home for a minimum of 8 weeks with the request for minimal use, a written log, and note-taking. After at least 8 weeks, the patients will return to the center or an OT will travel to their home for outcomes testing, and usage data from their prosthesis will be downloaded.

After the subjects have received training with either conventional or pattern recognition control, they will then return home for the initial 8 week home use trial. Subjects may also be provided with a laptop computer with a camera for Skyping. The laptop can be connected to the prosthesis, and the screen can be viewed remotely. The research team can then continue with remote follow up with more training and problem solving as needed.

Phase 2:

The subject will then return to one of the three centers for TMR surgery. Preoperative testing will include simple blood tests (approximately 10 cc or 2 teaspoons withdrawn) and may include other tests such as x-ray imaging or an electrocardiogram (ECG) to ensure the safety of the subject for surgery. If any other testing is needed, it will be discussed with the subject. The surgery method will involve the transfer of the median nerve to the *flexor digitorum superficialis* (DFS) muscle and the ulnar nerve will be transferred to the *flexor carpi ulnaris* (FCU) muscle. The median and ulnar nerves lie beside the target muscles, so they will be easy to transfer. Both transfers can be done with a single ventral incision. Alternative target muscles (e.g. the brachioradialis muscle) can be used if trauma precludes use of the DFS and/or FCU. Other subject-specific surgical procedures deemed appropriate by the study surgeon (e.g., neuroma revision, scar revision) will be discussed. After the surgery, the subject may be discharged home, or may remain in the hospital overnight and potentially longer to make sure that he or she is doing well. Some post-operative pain is expected and subjects will be given a prescription for pain medication if needed. The wound will need to be checked about one week after surgery and then again around three weeks after surgery. This can be done with the study surgeons remotely.

Subjects will return to using the prosthesis from Phase 1 with either conventional or pattern recognition control for 6 months after surgery. Socket adjustments will be made if needed. For the 6 subjects who return to using conventional control, this 6-month period will serve as a washout period so that any improvement in pattern recognition control after TMR is not simply due to continued practice and learned proficiency. For the 6 subjects who return to using pattern recognition control, we will track their usage and performance during the reinnervation period. These subjects may be provided with a laptop computer and asked to perform a series of virtual tests (e.g., a subset of our outcomes toolbox including Motion Test and TAC Test) a minimum of twice per month.

Six months after surgery, when reinnervation of the target muscles has been completed, all patients will return to using the prosthesis with pattern recognition control. Socket adjustments or replacement will be made, if needed. Subjects will then be retrained in pattern recognition control, only this time special attention will be paid to utilizing EMG from target muscles for intuitive control of additional functions. Preliminary testing will be done to ensure that subjects can adequately use the device. Subjects will then take the prosthesis home for a third 8-week home-use trial. Once again, they will be asked to log their daily use of the device and note any issues. After this home-use trial, the same comprehensive outcomes testing will be completed with the participants, and usage data from their prostheses will be downloaded.

Finally, subjects will continue using their prostheses at home for an additional 3-4 months. No usage requirements will be given to participants during this time. At the end of this 3-4 month period (approximately 12 months after surgery), the subjects will have a final round of testing with the complete toolbox and data from the prosthesis will be downloaded.

6.0 STATISTICAL PLAN

Sample size calculations were based on two sets of preliminary data (1) conventional and pattern recognition control performance by transhumeral and shoulder disarticulation TMR patients on the Box and Block Test; and (2) pre- and post-TMR conventional prosthesis control performance by transhumeral and shoulder disarticulation TMR patients on the Box and Block Test.

For all three data sets, the Type I error was set to α=0.05, and the Type II error was set to β=0.1 for a power of 0.9. Calculations were made based on a two-sided alternative hypothesis, since it is not yet evident that any of our proposed interventions will improve scores in the included outcome measures. Due to the small numbers of subjects anticipated in this study, a t-distribution was used instead of a z-distribution for sample size calculations [93, 94]. Sample size, *n*, was determined by solving the following equation:

$$\beta = T_{n-1}\left(t_{\alpha/2,n-1} \middle| \frac{|\delta|\sqrt{n}}{\sigma}\right)$$

where β is the Type II error, $T_{n-1}()$ is the cumulative distribution function of the noncentral *t*-distribution with n - 1 degrees of freedom, α is the Type I error, δ is the desired detectable difference, and σ is the population standard deviation (estimated by the between-subject sample standard deviation) [94].

Calculations for Conventional and Pattern Recognition Control Performance included performance on the Box and Block Test by one shoulder disarticulation subject and three transhumeral patients who had received TMR surgery. The average number of blocks moved in 2 minutes by the four subjects using conventional myoelectric control was 10.5 (SD 3.9). The average number of blocks moved in 2 minutes by the four subjects using pattern recognition control was 14.8 (SD 2.9). The average change in the number of blocks moved per subject was 4.3 (SD 5). A change of 3 blocks per minute, or δ = 6 blocks for the 2-minute task, has been determined to be clinically significant [95]. This gives an effect size of 6 ÷ 5 = 1.2, and a minimum sample size of 10 subjects, using the table provided in [94].

Calculations for Pre- and Post-TMR Performance included performance on the Box and Block Test by three shoulder disarticulation and three transhumeral subjects before and after TMR surgery. Subjects in both groups had control of the same prosthetic joints, and outcome assessment values were similar, so they were grouped together for this analysis. The average preoperative performance of the six subjects was 4.7 (SD 0.8) blocks in 2 minutes. The average post-operative result was 16.1 (SD 4.6) blocks in 2 minutes. The average change per person was 11.4 (SD 4.5) blocks. A change of 3 blocks per minute, or δ = 6 blocks for the 2-minute task, has been determined to be clinically significant [95]. This gives an effect size of 6 ÷ 4.5 = 1.33, and a minimum sample size of 9 subjects, using the table provided in [94].

Based on the power analysis of these three data sets, at least 10 subjects are required to complete the testing. In order to account for 40% attrition as well as to provide more robust data, 16 subjects will be tested in phase 1 and phase 2.

Subjects' performance will be compared using conventional control and pattern recognition control by completing a repeated measures analysis of variance (ANOVA) with the subject as the random factor, control style as the fixed factor, order in which they completed the

experiment (conventional first or pattern recognition first) as a fixed factor, and the subjects' average daily wear time will be included as a covariate.

This analysis will apply to the following metrics, calculated for all three specific aims: Modified Box and Block (number of blocks), Clothespin Relocation Test (time), ACMC (functional score), Jebsen Test of Hand Function (time for each task), SHAP (score for each prehensile pattern as well as overall score), OPUS–UEFS (functional score), AM-ULA (functional score), Motion Test (classification accuracy, motion completion rate, motion selection time, and motion completion time), TAC Test (classification accuracy, motion completion rate, motion completion time, path efficiency), PSFS (average difficulty rating) and usage data. A statistician will assist in or review our analyses to ensure appropriate execution and suggest any alternate or addition analyses once data has been collected.

7.0 SAFETY ASSESSMENT

7.1 RISK AND INJURY

The proposed surgical intervention is commonly performed. Pre-operative testing including blood draw may include the risk of momentary discomfort at the site of the blood draw, possible bruising, redness and swelling around the site. The risks primarily include the standard elective surgery risks on a limb, which include bleeding, bruising, infection, limb edema and complications of anesthesia. We expect minimal risk will be posed to these subjects during the procedure. There will be some swelling (edema) after surgery and drains may be used to reduce post-surgical swelling. This will temporarily prevent subjects from using his or her artificial limb. Wound healing should resolve in 2-4 weeks and limb swelling should resolve in 2-4 months allowing the subjects to get back to using a prosthesis after the swelling has gone down.

The risk of nerve transfer failure is very low—less than 6% based on cases to date and usually due to plexopathy or flap failure (neither of these issues will be relevant for below elbow amputees). Even if the nerve transfers were to fail, other myoelectric control sites would be available, thus subsequent prosthesis performance should not be impaired and patient function will not decline as a consequence of the procedure.

There is the potential that subjects may not initially perform well with the pattern recognition prosthesis, especially when trying to perform multiple hand grasps. In the beginning, a significant amount of time will be spent with the occupational therapist to promote optimal performance. However, we expect that each subject will be able to reliably perform at least two hand grasps, thus the device should have a baseline function similar to or better than a standard prosthesis. There is also potential that some subjects will not comply with the required prosthesis usage time during the home-use trial. Subjects who do not comply with the minimum usage time in the initial phase will be dropped from the study and will not proceed to surgery. By allowing for a 20% attrition rate over the course of all three specific aims, we hope to achieve adequate statistical results even if some subjects do not complete the entire study.

It is our hope that after participating in extensive preliminary testing and receiving TMR surgery, patients will carry through with this post-surgical testing. However, it is still possible that patient attrition will be an issue. Thus we are prepared for a 20% attrition rate after the TMR

surgery.

7.2 DATA COLLECTION & RECORD KEEPING

All data and medical information collected from subjects will be considered privileged and held in confidence: subjects will be assigned confidential codes and no identifying personal data will be stored with study data. Study data will be stored in password protected computers or in locked filing cabinets as applicable.

All recorded data will be stored on a password-protected computer at the SRALAB for further offline analysis. Each subject will be identified with a subject code, and filenames will be labeled with the subject code instead of subject name. The subject name - subject code link will be stored in a locked file cabinet at the SRALAB. In the case that the results of this data are published, the subjects will be referenced through the subject code and the identity of the research subjects will not be indicated. All consented subjects agree to photography and videotaping. If a subject gives specific permission, he/she may be photographed and videotaped during the experiment for possible use in scientific presentations or publications. If the subject consents to use of images/video in scientific presentations or publication, he/she will be informed that every effort will be made to protect subject privacy in these images, but they may include the face or other identifying features.

8.0 BENEFITS

The primary benefit in this research is to determine whether combining targeted muscle reinnervation surgery (TMR) and pattern recognition control—which together have substantially improved prosthesis control for higher-level amputees—provides similar benefits for individuals with below elbow amputation, thus both improving control of current state-of-the-art devices and providing impetus for development of even more functional devices.

9.0 COMPENSATION

The subjects will not have to pay any prosthetics or surgical costs for their participation in this study. All surgical and hospital fees related to the TMR surgery will be covered by the study sponsor.

Compensation for amputee patients will be $40.00 per hour up to $200 per day for each day of prosthesis training and testing plus the cost of lodging, travel, and parking if necessary for time spent in the laboratory as part of the testing. All travel costs including airfare, ground transportation, and lodging will be paid by SRALAB. Such arrangements will be facilitated by SRALAB staff. This has been our protocol for several years and seems to fairly relieve any financial burden and provide a modest incentive.

Travel and hotel costs are to be covered directly by SRALAB, other costs and daily rate to be reimbursed following each trip, per SRALAB travel policy. At the end of each visit, subjects will be sent a check or paid by Clincard for the number of days they were required to be on site for the study. For take-home use, subjects will not be held responsible if the prosthetic device stops working or is accidentally damaged during normal usage. In the event that subjects become unable to complete all components of the experiment, they will receive compensation for their involvement up to the point where they were unable to continue. In the event subjects decide to not complete all components of the study, they will be paid for all the prosthesis training and testing days they did participate, whether completing each task or not. Payment will not otherwise be prorated.

Following fulfillment of all study requirements including: all fitting, training and testing appointments, three 8 week at home trials with use of an average of 2 hours per day and weekly communication, and TMR surgery, subjects will be able to keep the prosthesis used in this study. If they need any follow up care or have maintenance issues with this prosthesis following the conclusion of this study, they will need to contact their current prosthetist. Maximal reimbursement is $3500 per subject for the prosthesis training and testing sessions. Subjects will not share in any future financial gain from the technology and/or devices licensed and/or patented by RIC.

Patients will be paid via check or ClinCard from the SRALAB. Payments in excess of $600 in a calendar year are required to be reported in taxes as income.

10.0 REFERENCES

[1] T. R. Dillingham*, et al.*, "Limb amputation and limb deficiency: Epidemiology and recent trends in the United States," *South Med J,* vol. 95, pp. 875-883, 2002.

[2] J. Davidson, "A survey of the satisfaction of upper limb amputees with their prostheses, their lifestyles, and their abilities," *J Hand Ther,* vol. 15, pp. 62-70, Jan-Mar 2002.

[3] G. Li*, et al.*, "Quantifying pattern recognition-based myoelectric control of multifunctional transradial prostheses," *IEEE Transactions on Neural Systems and Rehabilitation Engineering,* vol. 18, pp. 185-92, April 2010 2010.

[4] T. A. Kuiken*, et al.*, "The use of targeted muscle reinnervation for improved myoelectric prosthesis control in a bilateral shoulder disarticulation amputee.," *Prosthetics and Orthotics International,* vol. 28, pp. 245-253, December 2004.

[5] K. Ziegler-Graham*, et al.*, "Estimating the prevalence of limb loss in the United States: 2005 to 2050," *Archives of Physical Medicine and Rehabilitation,* vol. 89, pp. 422-429, Mar 2008.

[6] C. A. Krueger*, et al.*, "Ten years at war: comprehensive analysis of amputation trends," *J Trauma Acute Care Surg,* vol. 73, pp. S438-44, Dec 2012.

[7] P. F. Adams, *et al.*, "Current estimates from the National Health Interview Survey, 1996," *Vital Health Stat 10,* pp. 1-203, Oct 1999.
[8] M. S. Pinzur, *et al.*, "Functional outcome following traumatic upper limb amputation and prosthetic limb fitting," *Journal of Hand Surgery-American Volume,* vol. 19, pp. 836-839, 1994.
[9] D. J. Atkins, *et al.*, "Epidemiologic overview of individuals with upper-limb los and their reported research priorities," *Journal of Prosthetics and Orthotics,* vol. 8, pp. 2-11, 1996 1996.
[10] RSLSteeper. (April 5, 2013). *bebionic3 features*. Available: http://bebionic.com
[11] Touch Bionics. (April 5, 2013). *i-limb ultra*. Available: http://www.touchbionics.com/products/active-prostheses/i-limb-ultra/
[12] S. Schulz, "First experinces with the Vincent Hand," in *Myoelectric Controls/Powered Prosthetics Symposium*, Frederickton, New Brunswick, Canada, 2011.
[13] T. A. Kuiken, *et al.*, "The use of targeted muscle reinnervation for improved myoelectric prosthesis control in a bilateral shoulder disarticulation amputee," *Prosthetics and Orthotics International,* vol. 28, pp. 245-53, Dec 2004.
[14] T. A. Kuiken, *et al.*, "Targeted reinnervation for enhanced prosthetic arm function in a woman with a proximal amputation: a case study," *Lancet,* vol. 369, pp. 371-380, Feb 3 2007.
[15] L. A. Miller, *et al.*, "Improved Myoelectric Prosthesis Control Using Targeted Reinnervation Surgery: A Case Series," *Neural Systems and Rehabilitation Engineering, IEEE Transactions on [see also IEEE Trans. on Rehabilitation Engineering],* vol. 16, pp. 46-50, 2008.
[16] P. Zhou, *et al.*, "Decoding a New Neural-Machine Interface for Control of Artificial Limbs," *J Neurophysiol,* vol. 98, pp. 2974-82, Aug 29 2007.
[17] L. A. Miller, *et al.*, "Surgical and Functional Outcomes of Targeted Muscle Reinnervation," in *Targeted Muscle Reinervation: A Neural Interface for Artificial Limbs*, T. A. Kuiken, *et al.*, Eds., ed Boca Raton: CRC Press, 2013.
[18] T. A. Kuiken, *et al.*, "The Hyper-Reinnervation of Rat Skeletal-Muscle," *Brain Research,* vol. 676, pp. 113-123, APR 3 1995.
[19] R. D. Lipschutz, *et al.*, "Transhumeral level fitting and outcomes following targeted hyper-reinnervation nerve transfer surgery," in *Proceedings of the Myoelectric Control Symposium*, 2005, pp. 2-5.
[20] K. D. O'Shaughnessy, *et al.*, "Targeted Reinnervation to Improve Prosthesis Control in Transhumeral Amputees. A Report of Three Cases," *J Bone Joint Surg Am,* vol. 90, pp. 393-400, February 1, 2008 2008.
[21] M. Zecca, *et al.*, "Control of multifunctional prosthetic hands by processing the electromyographic signal," *Critical Reviews in Biomedical Engineering,* vol. 40, pp. 459-485, 2002.
[22] E. Scheme and K. Englehart, "EMG Pattern Recognition for the Control of Powered Upper Limb Prostheses: State-of-the-Art and Challenges for Clinical Use," *Journal of Rehabilitation Research and Development,* vol. 48, 2011.
[23] B. Hudgins, *et al.*, "A new strategy for multifunction myoelectric control," *IEEE Transactions on Biomedical Engineering,* vol. 40, pp. 82-94, Jan 1993.

[24] Y. H. Huang, *et al.*, "A Gaussian mixture model based classification scheme for myoelectric control of powered upper limb prostheses," *IEEE Transactions on Biomedical Engineering,* vol. 52, pp. 1801-1811, NOV 2005.

[25] O. Fukuda, *et al.*, "EMG-based Human-Robot Interface for Rehabilitation Aid," in *Proceedings of the 1998 IEEE International Conference on Robotics and Automation*, Leuven, Belgium, 199.

[26] P. J. Gallant, *et al.*, "Feature-based classification of myoelectric signals using artificial neural networks," *Medical & Biological Engineering & Computing,* vol. 36, pp. 485-489, JUL 1998.

[27] D. Graupe, *et al.*, "Multifunctional Prosthesis and Orthosis Control Via Microcomputer Identification of Temporal Pattern Differences in Single-Site Myoelectric Signals," *Journal of Biomedical Engineering,* vol. 4, pp. 17-22, 1982.

[28] S. H. Park and S. P. Lee, "EMG pattern recognition based on artificial intelligence techniques," *IEEE Transactions on Rehabilitation Engineering,* vol. 6, pp. 400-405, 1998.

[29] J. S. Han, *et al.*, "New EMG pattern recognition based on soft computing techniques and its application to control a rehabilitation robotic arm," in *International Conference on Soft Computing*, Fukuoka, Japan, 2000, pp. 1-4.

[30] F. H. Y. Chan, *et al.*, "Fuzzy EMG classification for prosthesis control," *IEEE Transactions on Rehabilitation Engineering,* vol. 8, pp. 305-311, SEP 2000.

[31] A. B. Ajiboye and R. F. Weir, "A heuristic fuzzy logic approach to EMG pattern recognition for multifunctional prosthesis control," *IEEE Transactions on Neural Systems and Rehabilitation Engineering,* vol. 13, pp. 280-291, Sep 2005.

[32] L. Hargrove, *et al.*, "A Comparison of Surface and Intramuscular Myoelectric Signal Classification," *IEEE Transactions on Biomedical Engineering,* vol. 54, pp. 847-853, May 2007.

[33] A. Simon, *et al.*, "Target Achievement Control Test: Evaluating real-time myoelectric pattern-recognition control of multifunctional upper-limb prostheses " *Journal of Rehabilitation Research and Development,* vol. 18, pp. 619-628, 2011.

[34] A. Simon, *et al.*, "A decision-based velocity ramp for minimizing the effect of misclassifications during real-time pattern recognition control," *IEEE Transactions on Biomedical Engineering,* vol. 58, pp. 2360 - 2368, 2011.

[35] B. A. Lock, *et al.*, "Prosthesis-guided training for practical use of pattern recognition control of prostheses," in *Myoelectric Controls/Powered Prosthetics Symposium*, Fredericton, New Brunswick, Canada, 2011.

[36] P. Kyberd, "Advances in Upper Limb Prosthetics - MEC' 11," *Journal of Prosthetics and Orthotics,* vol. 24, pp. 54-55, 2012.

[37] L. Hargrove, *et al.*, "Pattern Recognition Control Outperforms Conventional Myoelectric Control in Upper Limb Patients with Targeted Muscle Reinnervation," in *Proceedings of the 35th International Conference of the IEEE Engineering in Medicine and Biology Society (EMBS)*, Osaka, Japan, 2013, pp. 1599-1602.

[38] T. A. Kuiken, *et al.*, "Targeted muscle reinnervation for real-time myoelectric control of multifunction artificial arms," *JAMA,* vol. 301, pp. 619-28, Feb 11 2009.

[39] H. Huang, *et al.*, "An Analysis of EMG Electrode Configuration for Targeted Muscle Reinnervation Based Neural Machine Interface," *IEEE Transactions on Neural Systems and Rehabilitation Engineering,* vol. 16, pp. 37-45, 2008.
[40] G. Kvas and R. Velik, "A Filter approach for myoelectric channel selection," in *Industrial Informatics, 2008. INDIN 2008. 6th IEEE International Conference on*, 2008, pp. 1437-1440.
[41] R. A. Bueno, *et al.*, "Targeted Muscle Reinnervation of a Muscle-Free Flap for Improved Prosthetic Control in a Shoulder Amputee: Case Report," *Journal of Hand Surgery-American Volume,* vol. 36A, pp. 890-893, May 2011.
[42] P. S. Kim, *et al.*, "The effects of targeted muscle reinnervation on neuromas in rabbit rectus abdominis flap model," *Journal of Hand Surgery. American Volume,* vol. 37, pp. 1609-1616, 2012.
[43] J. H. Ko, *et al.*, "Targeted Muscle Reinnervation as a Strategy for Neuroma Prevention," in *Targeted Muscle Reinnervation: A Neural Interface for Artificial Limbs*, T. A. Kuiken, *et al.*, Eds., ed Boca Raton: CRC Press, 2013.
[44] D. Tkach, *et al.*, "Myoelectric Control Performance Provided by Generic Electrode Grid When Used with Targeted Muscle Reinnervation Patients," in *Proceedings of the 34th International Conference of the IEEE Engineering in Medicine and Biology Society (EMBS)*, San Diego, 2012.
[45] A. Young, *et al.*, "Improving Myoelectric Pattern Recognition Robustness to Electrode Shift by Changing Interelectrode Distance and Electrode Configuration," *IEEE Transactions on Biomedical Engineering,* vol. 59, pp. 645-652, 2012.
[46] A. Simon, *et al.*, "Patient Training for Functional Use of Pattern Recognition-Controlled Prostheses," *Journal of Prosthetics and Orthotics,* vol. 24, pp. 56-64, 2012.
[47] L. Hargrove, *et al.*, "A Comparison of Surface and Intramuscular Myoelectric Signal Classification," *IEEE Transactions on Biomedical Engineering,* vol. 54, pp. 847-853, 2007.
[48] L. H. Smith, *et al.*, "Determining the optimal window length for pattern recognition-based myoelectric control: balancing the competing effects of classification error and controller delay," *IEEE Transactions on Neural Systems and Rehabilitation Engineering,* vol. 19, pp. 186-192, 2011.
[49] A. Simon, *et al.*, "A Comparison of Proportional Control Methods for Pattern Recognition Control," in *33rd International Conference of the IEEE Engineering in Medicine and Biology Society*, Boston, MA, USA, 2011, pp. 3354-3357.
[50] J. A. Birdwell, *et al.*, "Activation of individual extrinsic thumb muscles and compartments of extrinsic finger muscles," *J Neurophysiol,* vol. 110, pp. 1385-92, Sep 2013.
[51] J. A. Birdwell, *et al.*, *Extrinsic Finger and Thumb Muscles Command a Virtual Hand to Allow Individual Finger and Grasp Control,* vol. Under Minor Revision.
[52] A. Young, *et al.*, "Classification of Simultaneous Movements using Surface EMG Pattern Recognition," 2012.
[53] T. R. Farrell and R. F. Weir, "The optimal controller delay for myoelectric prostheses," *IEEE Trans Neural Syst Rehabil Eng,* vol. 15, pp. 111-8, Mar 2007.
[54] S. C. Chow, *et al.*, "A note on sample size calculation for mean comparisons based on noncentral t-statistics," *J Biopharm Stat,* vol. 12, pp. 441-56, Nov 2002.

[55] W. Hill*, et al.*, "Functional outcomes in the WHO-ICF model: Establishment of the Upper Limb Prosthetic Outcome Measures Group," *Journal of Prosthetics and Orthotics,* vol. 21, pp. 275-294, 2009.
[56] K. Stubblefield and T. A. Kuiken, "Occupational Therapy for the Targeted Muscle Reinnervation Patient," in *Targeted Muscle Reinnervation: A neural interface for artificial limbs*, T. Kuiken*, et al.*, Eds., ed Boca Raton: CRC Press, 2013, pp. 99-118.
[57] V. Mathiowetz*, et al.*, "Adult norms for the box and blocks test of manual dexterity," *American Journal of Occupational Therapy* vol. 39, pp. 386-391, 1985.
[58] C. Light*, et al.*, "Establishing a Standardized Clinical Assessment Tool of Pathologic and Prosthetic Hand Function: Normative Data, Reliability, and Validity," *Archives of Physical Medicine and Rehabilitation,* vol. 83, pp. 776-783, 2002.
[59] R. H. Jebsen*, et al.*, "An objective and standardized test of hand function," *Arch Phys Med Rehabil,* vol. 50, pp. 311-9, Jun 1969.
[60] L. M. Hermansson*, et al.*, "Assessment of capacity for myoelectric control: a new Rasch-built measure of prosthetic hand control," *J Rehabil Med,* vol. 37, pp. 166-71, May 2005.
[61] H. Y. Lindner*, et al.*, "Assessment of capacity for myoelectric control: evaluation of construct and rating scale," *J Rehabil Med,* vol. 41, pp. 467-74, May 2009.
[62] H. Burger*, et al.*, "Validation of the orthotics and prosthetics user survey upper extremity functional status module in people with unilateral upper limb amputation," *J Rehabil Med,* vol. 40, pp. 393-9, May 2008.
[63] P. Stratford*, et al.*, "Assessing disability and change on individual patients: a report of a patient specific measure," *Physiotherapy Canada,* vol. 47, pp. 258-263, 1995.
[64] K. A. Stubblefield*, et al.*, "Occupational therapy outcomes with targeted hyper-reinnervation nerve transfer surgery: Two case studies," in *MyoElectric Controls/Powered Prosthetics Symposium*, Fredericton, New Brunswick, Canada, 2005.
[65] A. J. Young*, et al.*, "A comparison of the real-time controllability of pattern recognition to conventional myoelectric control for discrete and simultaneous movements," *Journal of Neuroengineering and Rehabilitation,* vol. 11, Jan 10 2014.
[66] A. J. Young*, et al.*, "Classification of Simultaneous Movements Using Surface EMG Pattern Recognition," *IEEE Transactions on Biomedical Engineering,* vol. 60, pp. 1250-1258, May 2013.
[67] S. M. Wurth and L. J. Hargrove, "A real-time comparison between direct control, sequential pattern recognition control and simultaneous pattern recognition control using a Fitts' law style assessment procedure," *Journal of Neuroengineering and Rehabilitation,* vol. 11, pp. 91-91, 2014 May 2014.
[68] H. Huang*, et al.*, "Spatial Filtering Improves EMG Classification Accuracy Following Targeted Muscle Reinnervation," *Annals of Biomedical Engineering,* vol. 37, pp. 1849-1857, 2009.
